# Effect of chromium doping on the band gap tuning of titanium dioxide thin films for solar cell applications

**DOI:** 10.1016/j.heliyon.2023.e23096

**Published:** 2023-11-30

**Authors:** Abdul Ahad, Jiban Podder, Tusar Saha, Hari Narayan Das

**Affiliations:** a Department of Physics, Bangladesh University of Engineering and Technology, Dhaka, 1000, Bangladesh; b Department of Physics, Comilla University, Comilla, 3506, Bangladesh; c American International University-Bangladesh, Dhaka, 1229, Bangladesh; d Bangladesh Atomic Energy Center Dhaka, Dhaka, 1000, Bangladesh

**Keywords:** Spray pyrolysis, Cr doped TiO_2_ thin films, Surface morphology, Anatase-rutile-mixed phase, Band gap, Resistivity

## Abstract

A simple and inexpensive spray pyrolysis deposition (SPD) approach was used to produce TiO_2_ and Cr (2–8) at.%-doped TiO_2_ thin films. To explore the morphological features of the films, FE-SEM micrographs were used and found that 6 and 8 at.% TiO_2_:Cr films had fibrous patterns with diameters of 0.45 and 0.78 μm, respectively, while the remainder of the films were agglomerated particles. From X-ray diffraction investigation, it was found that the TiO_2_ thin films had an anatase crystal phase (tetragonal) up to 6 at.% Cr doping, while an anatase-rutile mixed crystalline phase was identified for 8 at.% Cr doping. The crystallite size of the pristine TiO_2_ film was 35 nm, while for TiO_2_:Cr films, it ranges from 35 to 46 nm. The Fizeau fringes technique was employed to measure the thickness of the TiO_2_ film and 165 nm was found for pristine TiO_2_ and 164–180 nm for TiO_2_:Cr films. UV–visible spectroscopy was used to study optical properties such as absorbance, refractive index, optical band gap, dielectric constant, and optical conductivity. As the Cr concentration increases, the optical band gap decreases from 3.40 eV to 2.70 eV. Using the four-point probe method, it was found that the resistivity changes with temperature and is also affected by the Cr content.

## Highlights

• We reported the modified structure of TiO_2_ by doping Cr. Substituting Cr^3+^ in place of Ti^4+^ in TiO_2_ lattice transformed n to p-type semiconductor.
• Cr inclusion effectively tailored the band of TiO_2_:Cr thin films and change their anatase-to-rutile phase transition.
• XRD data shows the TiO_2_ thin films an anatase phase up to 6 at.% Cr, while an anatase-rutile mixed phase for 8 at.% Cr doping.
• The band structure and resistivity of the films make them potential candidates in the manufacturing of optical devices.

## 1 Introduction

Energy consumption is increasing day by day in this competitive era. Hence, human dependence on renewable energy is essential. In recent years, the development of optical and electrical energy storage devices using nanoparticles, nanoplates, and nanocomposite films has attracted widespread attention [[1], [2], [3]]. These materials have unique properties and advantages that can improve the performance and efficiency of energy storage systems such as solar cells, which play an important role in our daily lives. Many researchers have proposed that titanium dioxide (TiO_2_)-based solar cells can be more efficient than those that exist in the present world, but they are still not successfully marketable [[4], [5], [6]]. An abundantly utilized semiconductor material is TiO_2_, which is employed in photovoltaic devices, gas sensors, electrochromic displays, photocatalyst applications [7], smart windows [8], antireflection coatings [9], optical filters [10], and dye-sensitized solar cells [11,12], etc. Due to its certain special behaviors like environmentally friendly nature, easy synthesis ways and optical band gap tuning by doping and inoculating with visible light absorbing dyes. Based on crystallinity and band gap values, TiO_2_ can exist in three different allotropies, like anatase, rutile, and brookite forms. The most prevalent phases used in solar cell applications are anatase and rutile, and their commercially accessible optical band gaps (E_gap_) are 3.2 and 3.0 eV, respectively [13].

A considerably higher E_gap_ (3.2) that exists in the ultraviolet area of the solar spectrum can be modified by doping TiO_2_ with a transition metal (such as Cr, Fe, and Ni), which is one of the most effective strategies for sensitizing TiO_2_ to visible light and for creating charge traps to retain electron-hole pairs isolate [14]. In photovoltaics, the capacity to control the E_gap_ of the absorbing films is essential to making the most of the solar spectrum. Furthermore, high-efficiency photovoltaic devices require a comprehensive understanding of the optical characteristics of the absorbing passive or active oxide layer, such as refractive index and extinction coefficient. Many articles have been published on defect formation and its influence on the optical and electrical characteristics of the TiO_2_ lattice [15,16]. These defects played a vital role in modifying the characteristics of the thin films in different ways that depend highly on the growth routes and deposition conditions.

Previous studies have explored the properties of Cr-doped TiO_2_ films using various methods, but limited attention has been paid to TiO_2_:Cr thin films produced through the Spray Pyrolysis Deposition (SPD) technique to investigate their structural and optical properties as well as electrical activation energy. Mardare et al. studied SPD grown TiO_2_:Cr thin films. However, the details of the optical and electrical properties of the films on glass substrates were not explored [17]. Wang et al. performed low-temperature electrical observations up to 300 K [18], while the present investigation focuses on a wider temperature range, extending beyond room temperature to nearly 393 K. This wider temperature range allows us to gain insights into the semiconducting properties of TiO_2_:Cr films. Additionally, the previous article did not discuss the long-term stability and durability of TiO_2_:Cr films, which are very important for practical applications. Notably, our non-vacuum preparation technique makes our samples less sensitive to atmospheric conditions, thus contributing to their relative stability over time.

Due to the nearly equal ionic radius of Cr^3+^ (0.69 Å) and Ti^+4^ (0.68 Å), they are easily replaceable [19] and Cr^3+^ ions can go into the lattice as substitution metal dopant. The incorporation of Cr in TiO_2_ lattice does not affect the crystallography of pure TiO_2_ material and trivalent Cr acts as an acceptor type impurity which can be written by the following equation [20]:

$${Cr}_{2}O_{3}\to {2Cr}_{Ti}+V_{O}+{3O}_{\dot{O}}$$

where V_o_ is the oxygen vacancies and ${\text{Cr}}_{\text{Ti}}$ is the Cr substitution in Ti sites. Pristine and doped TiO_2_ thin films can be synthesized using different techniques, including sol-gel, pulsed laser deposition (PLD), RF magnetron sputtering, electrodeposition, coating, hydrothermal, chemical bath deposition (CBD), chemical vapor deposition (CVD) [21], and spray pyrolysis deposition (SDP) [22]. Among them, SPD technique is mostly used due to its low-cost, non-vacuum chamber and ease of control of the films' compositional parameters and uniform growth over large area substrates [23,24].

It is important to remember that Cr doping creates energy levels within the TiO_2_ lattice and the band gap of the low dopant (2–8) at.% concentrations are preferred to avoid excessive distortion of the semiconductor’s electronic structure, which also tunes the band gap slightly, making it more suitable for solar absorption without dramatically altering the material’s properties and reduces recombination of charge carriers by minimizing defects for maximizing efficiency. Furthermore, the presence of an anatase-rutile mixed phase (after 6 at.% Cr doping) restricts us from exceeding a dopant level of 8 at.%.

In this study, we have tried to modify the structure of TiO_2_ semiconductors by incorporating Cr using the SPD technique. Substituting Cr^3+^ in place of Ti^4+^ in the TiO_2_ lattice may be converted from n-type to a p-type semiconductor, and Ti^4+^ ion reacts with the Cr^3+^ ion with a compensating charge imbalance. Cr^3+^ ion acts as an acceptor in the TiO_2_ lattice and creates oxygen vacancies that are responsible for defect generation in the valence to the conduction band. It has been discovered that the Cr inclusion not only enables the creation of TiO_2_:Cr thin films to effectively tailor their bandgap but also initiates their anatase-to-rutile phase transition. This modification of the band structure and resistivity of the films makes them effective candidates for the manufacturing of optical devices like solar cells.

## 2 Experimental details

### 2.1 Solution preparation

Reagent-grade titanium butoxide [Ti(OC_4_H_9_)_4,_ (97%)] and chromium chloride hexahydrate [CrCl_3_.6H_2_O, (96%)] (Company: Sigma Aldrich) were used as precursor materials of Ti and Cr atom. Pristine TiO_2_ and TiO_2_:Cr thin films were deposited onto the transparent glass substrates by the SPD technique. To prepare a pristine TiO_2_ thin film, 3.40 g of Ti(OC_4_H_9_)_4_ was mixed into 80 mL distilled water and 20 mL ethanol that confirmed 0.1 M concentrated solution, then added approximately 0.75 mL HCl in the solution to make it completely aqueous. For the preparation of 2, 4, 6, and 8 at.% TiO_2_:Cr solution, 0.0533, 0.1066, 0.1599, and 0.2132 g CrCl_3_.6H_2_O and 3.3354, 3.2671, 3.199, and 3.1309 g Ti(OC_4_H_9_)_4_ were mixed with 80 mL distilled water and 20 mL ethanol, and a similar amount of HCl was added that was used for pristine TiO_2_ film. Each solution was stirred for about 2 h in a magnetic stirrer and then filtered to get a homogeneous solution.

### 2.2 Deposition of pristine TiO_2_ and TiO_2_:Cr thin films

**Fig. 1:**
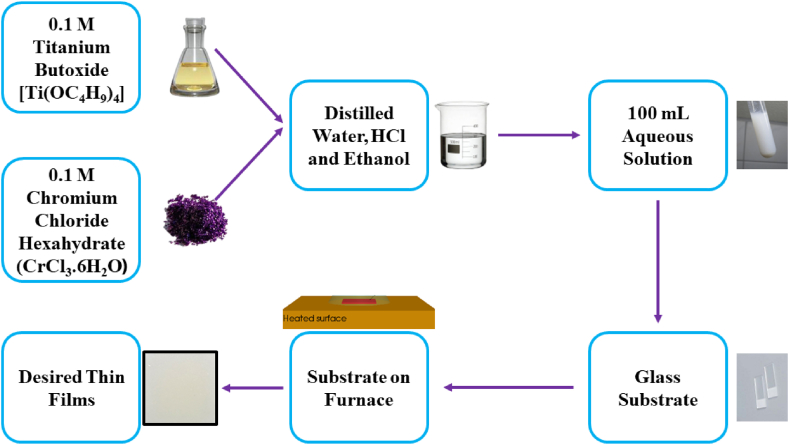
Flow chart of the synthesized process of TiO_2_:Cr thin films.

Plain transparent glass substrates were cut into 2.5 × 2.5 cm ratios using a diamond cutter and cleaned ultrasonically for 10 min using water, acetone, and ethanol solvent. Then these substrates were put on the mask and positioned in the furnace. Each time one substrate was used in the mask. Our deposition chamber was non-vacuum. We used an optical pyrometer to confirm the reaction temperature of 450 °C. Table 1 shows the conditions of deposited TiO_2_ and TiO_2_:Cr thin films whereas Fig. 1 shows the flow chart of the synthesis process. At the time of the deposition of the films on the glass substrate, some chemical reactions (Eqs. (1), (2), (3))) may undergo between the precursor materials. The reaction that is involved between Ti (IV)-butoxide and water molecules for pristine TiO_2_ thin film is given as follows:

(1) $$Ti{\left(OC_{4}H_{9}\right)}_{4}+4H_{2}O\to Ti{\left(OH\right)}_{4}+4C_{4}H_{9}OH$$

**Table 1:** The synthesized conditions during the deposition of pristine TiO_2_, and TiO_2_:Cr thin films.

Sl no.	Deposition parameters	Value/Grade	
			TiO_2_:Cr
1.	Synthesized temperature		450 °C
2.	Synthesized time		10±1 min
3.	Molar concentration		0.1 M
4.	Doping percentage		0.0–8.0 at.%
5.	Substrate to nozzle distance		25 cm
6.	Air flow pressure		0.5 bar
7.	Amount of solution		100 mL
8.	Rate sprayed solution		5 mL/min

TiO_2_ is produced because of the hydrolyzed species condensing:

(2) $$Ti{\left(OH\right)}_{4}\;\to TiO_{2}+2H_{2}O$$

TiO_2_:Cr thin films were formed following the reactions:

(3) $$\left(1-x\right)Ti{\left(OC_{4}H_{9}\right)}_{4}+x{CrCl}_{3}.6H_{2}O+2H_{2}O+\left(4-\frac{15x}{4}\right)O_{2}\Delta \to \;{Cr}_{x}{Ti}_{1-x}O_{2}+10\left(1-x\right)CH_{4}↑+6\left(1-x\right)CO_{2}↑+3xHCl↑+\frac{13x}{2}H_{2}O↑$$

By the Fizeau fringes technique [25,26], thicknesses of the synthesized films were investigated and found at 165, 180, 164,175, and 170 nm.

### 2.3 Characterizations

The as-deposited pristine TiO_2_ and TiO_2_:Cr thin films were subjected to their structural, optical, and electrical characterizations by different instrumental tools. The morphological properties of the films were investigated by field emission scanning electron microscopy (JEOL JSM-7600F). Structural investigation of the synthesized films was completed by an X-ray diffractometer (Model: Philips PANalytical X’PERT-PRO) CuK_α_ radiation (λ = 1.541 Å). From XRD data, the crystalline form of the films, lattice parameters, dislocation density, crystallite size, and lattice strain were evaluated. Vesta (open source) software was used to draw the crystal phase of anatase and rutile TiO_2_ semiconductors. The absorbance spectra of the films were measured using an Ultraviolet–Visible–NIR spectrophotometer (UV–Vis) in the 300–1000 nm photon wavelength range. Analyzing absorption data, various optical parameters, like absorbance, refractive index, optical band gap, and optical conductivity were calculated. A very common and cost-effective 4-point probe method was employed to determine the resistivity and activation energy of the films.

### 2.4 Morphological study

**Fig. 2:**
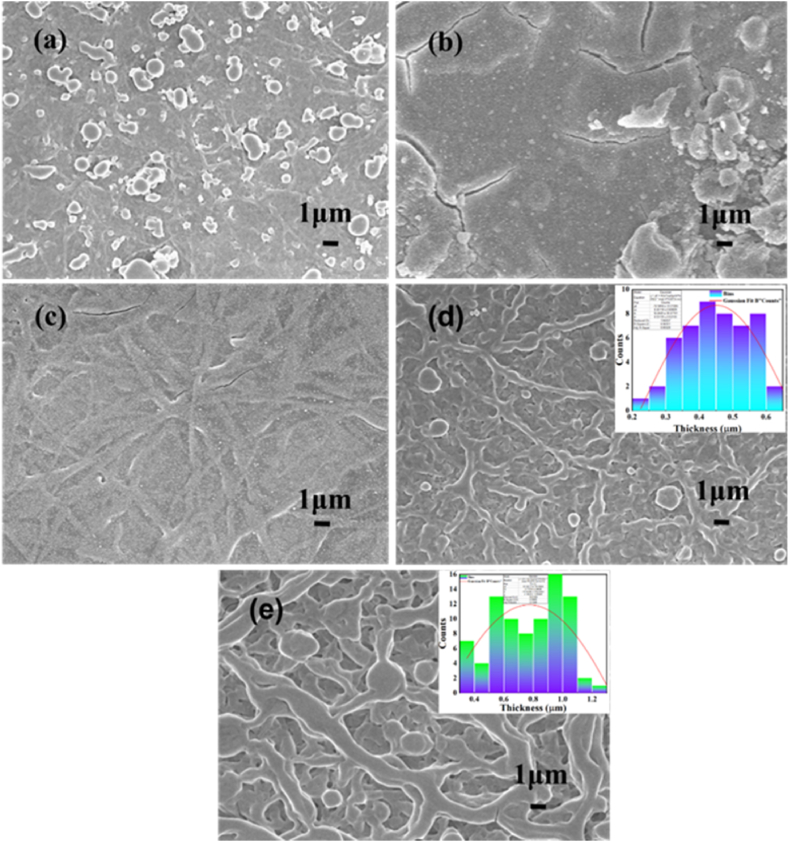
FE-SEM images of (a) pristine TiO_2_, (b) 2 at.%, (c) 4 at.%, (d) 6 at.%, and (e) 8 at.% TiO_2_:Cr thin films captured at 5k magnification.

Observing a material’s surface morphology is a crucial parameter for understanding its optical and electrical properties. The morphological characteristics of pristine TiO_2_ and TiO_2_:Cr thin films were examined using FE-SEM, and these FE-SEM micrographs are shown in Fig. 2. It is noticed that the substrate is consistently covered with a regular matter of agglomeration of the grain distribution (Fig. 2(a and b)) and fibers shape (Fig. 2(c–e)). Mechiakh et al. claimed that agglomeration develops in the deposited films when the kinetic energy is insufficient to persuade the grains to form [27]. Besides, when the spray droplets collide with the surface of the substrate, they may be dried, and due to this phenomenon, the droplets may not get enough time to spread out on the surface uniformly, and as a result, a rough surface is grown [28].

In the micrographs of 2 and 4 at.% TiO_2_:Cr films (Fig. 2(b and c)) some cracks are present. Several parameters are included to form a crack in the as-deposited films, like decreasing bonding strength between TiO_2_ lattice and Cr content when the film is very thick, an impact of capillary forces that are created due to the rapid evaporation of solvents from the surface of the film at the time of the drying process, and a mismatch of the thermal stretch between the glass substrate and the TiO_2_:Cr thin films [29].

It is seen that with doping, the agglomeration is gradually retarded, and the growth of grain is accelerated toward the fibrous formation. In the 6 and 8 at.% TiO_2_:Cr films, fibers are observed with diameters 0.45 and 0.78 μm as determined by ImageJ software (open source) which is shown in the inset of Fig. 2(d and e). In the case of 4 at.% Cr doped film (Fig. 2(c)), fiber structure is started initially to grow. The fibrous pattern observed in TiO_2_:Cr thin films can be attributed to various factors related to the deposition process like, deposition parameters, solvent evaporation and crystallization, surface tension, capillary effects, agglomeration and nucleation, thermal effects, and the viscoelastic properties of the material itself. In nature, viscoelastic materials are made up of long, flexible fiber-like particles. Materials that exhibit viscoelastic properties can be considered as having both elastic and viscous components under the applied stress causes an elastic strain right away. Owing to their shape of the particle and flexibility, the particle can easily glide along each other and momentarily connect to other to form a long chain fiber due to the fluid properties. As water is a polar solvent and has a high dielectric constant that causes the attenuation of electrostatic repulsion. The reduced density of charges causes a fiber network to be disrupted into droplets, leading to the formation of beaded fibers.

The development of fiber-like structures in TiO_2_:Cr could potentially be linked to its viscoelastic properties. Substances with viscoelastic attributes can be characterized by their simultaneous possession of both elastic and viscous elements, resulting in an immediate elastic deformation when subjected to applied stress. This is facilitated by the particles' shape and flexibility, allowing them to effortlessly slide against one another and temporarily link with adjacent particles. As a result of this fluid-like quality, these momentary connections lead to the creation of extended chain-like fibers. These particles to fiber nature have been found and explain it in detail [30]. Previously Taskin et *al.* found the nanofibers on the glass substrate for Zn doped cobalt oxide films [31] and Van-Pham et al., found fiber shape images in the case of Fe-doped TiO_2_ nanoparticles [32].

### 2.5 Elemental evaluation

**Fig. 3:**
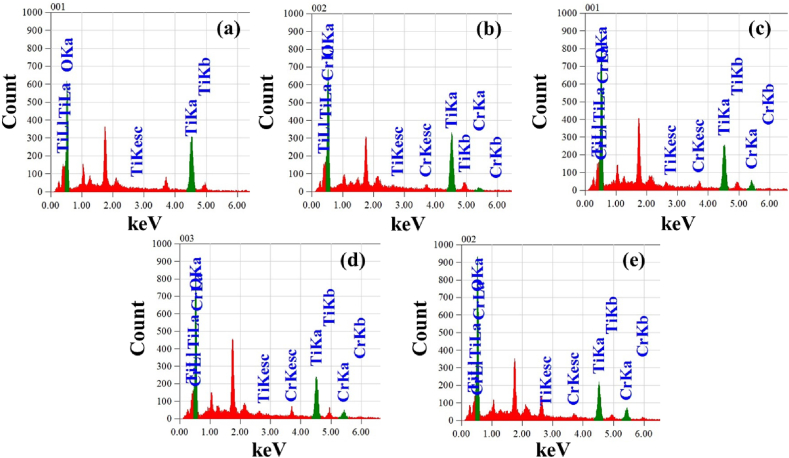
EDX micrographs of (a) pristine TiO_2_, (b) 2 at.%, (c) 4 at.%, (d) 6 at.%, and (e) 8 at.% TiO_2_:Cr thin films at 450 °C temperature and accelerating voltage 5 kV.

Energy Dispersive X-ray Spectroscopy (EDX) was employed to analysis the presence of elements in the pristine TiO_2_ and TiO_2_:Cr films and Fig. 3 shows the elemental peaks of EDX spectra of all the films. The deposited films are found to be stoichiometric, and the EDX analysis of pristine TiO_2_ film (Fig. 3(a)) affirms the presence of titanium atoms (Ti) and oxygen atoms (O). Pristine TiO_2_ film consists of Ti and O of about 24.61 & 75.39 at.%, and 49.38 & 50.62 mass%, respectively, which are visible by two strong peaks. In Fig. 3(b–e) one small extra peak denoted by the Ti–K emission line is found that represents the presence of the Cr atom in the TiO_2_ lattice.

Table 2 describes the atomic percentages (at.%) and mass percentages (mass%) of TiO_2_:Cr thin films whereas an indicative of the uniform integration of Cr atoms into the TiO_2_ lattice. Moreover, there is no trace of any other impurities within the detection limit of EDX.

**Table 2:** Statistical evolution of EDX outcomes of the films.

TiO_2:_Cr (x)	Element, at.%			Element, mass%		
	Ti	O	Cr	Ti	O	Cr
0	24.61	75.39	–	49.38	50.62	–
2	24.25	73.86	1.89	47.58	48.40	4.02
4	18.72	75.32	5.91	37.19	49.97	12.83
6	18.18	75.64	6.18	36.20	50.41	13.39
8	15.66	75.47	8.87	31.00	49.93	19.7

## 3 Structural analysis

### 3.1 X-ray diffractometer

**Fig. 4:**
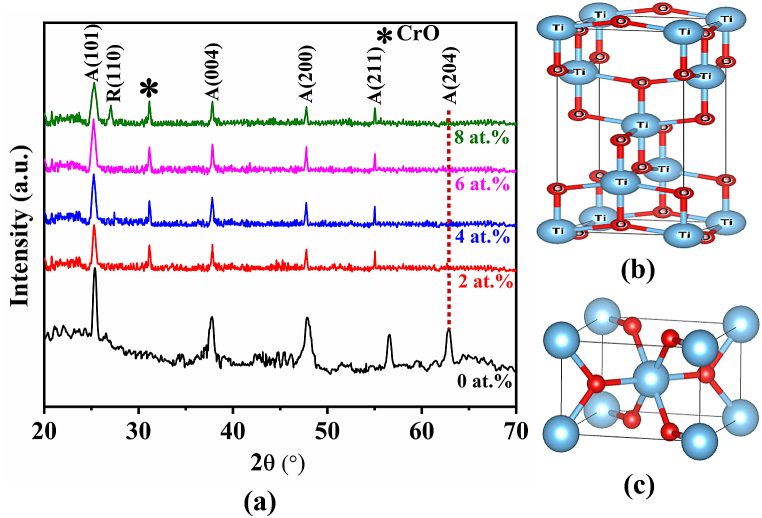
(a) XRD patterns of pristine TiO_2_ and TiO_2_:Cr thin films deposited onto the glass substrate, (b) anatase and (c) rutile crystal phase of TiO_2_ semiconductor.

X-ray diffractograms (XRD) are an important feature for assessing a material’s crystallinity and phase identification. They also provide information about how the actual structure differs from the ideal structure due to the incorporation of dopants into the crystal lattice. Fig. 4(a) depicts the XRD spectra of the pristine TiO_2_ and TiO_2_:Cr thin films grown on the glass substrates. In the spectrum, the anatase structure is denoted by ‘A’, and the rutile is by ‘R'. The diffracted peaks (101), (004), (200), (211), and (204) have been found for the pristine TiO_2_ at different angles. In addition, one impurity peak has been observed on 2 to 8 at.% Cr doping in the TiO_2_ lattice. The impurity peak is presented by a star (⁎) which may be appeared due to CrO [33]. Moreover, an anatase-to-rutile phase transition has been observed in the 8 at.% TiO_2_:Cr thin film due to the higher concentration of Cr incorporation [34]. The indexing patterns are comparable to the prior results [33].

According to the findings, all the films have a tetragonal crystal structure, and the crystallites exhibit the anatase phase with a preferential orientation of (101) plane direction. The intensities of the diffraction peaks are reduced by doping which may be related to the partial crystallization of TiO_2_. It is seen that Cr concentrations up to 6 at.% stabilize the orientation with preferential growth in the (101) plane direction and increase the intensity of the (101) peak, suggesting an improvement in the films' anatase phase crystallinity. At 8 at.% Cr doped TiO_2_ film, anatase and rutile mean mixed phase is found as well as the peak intensity of (101) direction is decreased. These results demonstrate that Cr ions may either systematically substitute Ti ions without changing the host TiO_2_ matrix or maybe remain dissoluble as Cr interstitial in the TiO_2_ matrix [35]. However, to confirm this alleged concept, more research is necessary.

With an increase in Cr content, the anatase peaks are shifted slightly (Table 3), which correlates with a decrease in the unit cell parameter ratio (c/a). In the case of 8 at.% TiO_2_:Cr film, due to anatase rutile mixed phase the peak shifting are no longer to lower diffraction angle that makes slightly large value of (c/a). The formation of oxygen vacancies is caused by the presence of more Cr atoms in the TiO_2_ lattice which might facilitate the anatase-rutile mixed phase to better fit the excess of Cr ions into the TiO_2_ lattice [36]. Several previous studies demonstrated that ions at substitutional sites with valency less than four and a smaller radius (e.g., Li^+^, Cr^3+^, Fe^3+^, Mn^2+^, Co^2+^, and Cu^2+^) even at mmol% concentration, can rise the oxygen vacancies caused by the transformation of anatase-to-rutile phase [37,38]. However, some of the TiO_2_ peaks have vanished as a result of the glass substrate’s amorphous character. Fig. 4(b and c) shows the crystalline structure of the anatase and rutile TiO_2_ phase that is drawn by vesta software (open source).

**Table 3:** The value of a = b, c, FWHM, V, c/a, D_s_, δ, and *ε* of pristine TiO_2_ and TiO_2_:Cr thin films.

Cr (at.%)	Lattice parameter		FWHM	V(Å)^3^	c/a	D_s_ (nm)	δ $\times 10^{14}$ lines/m^2^	*ε*$\times 10^{-4}$
	a = b (Å)	c (Å)						
0	3.7823	9.4118	0.4298	134.64	2.4883	35	8.16	9.81
2	3.7974	9.5129	0.3677	137.18	2.5051	46	4.71	8.42
4	3.7981	9.5137	0.3845	137.24	2.5049	42	5.62	8.83
6	3.8057	9.5131	0.3960	137.78	2.4997	41	5.78	9.13
8	3.7854	9.5139	0.3847	136.33	2.5133	35	8.03	8.84

### 3.2 Lattice parameters and crystallite size

Lattice constants or lattice parameters are the physical dimensions of the unit lengths along each crystallographic axis and their interaxial angles that determine the geometry of the unit cells in a crystalline lattice. Eq. (4) was applied to calculate the lattice parameters of the tetragonal anatase and rutile crystal structures of pristine TiO_2_ and TiO_2_:Cr films.

(4) $$\frac{1}{d_{hkl}^{2}}=\frac{h^{2}+k^{2}}{a^{2}}+\frac{l^{2}}{c^{2}}$$

where d_hkl_ is the interplanar distance, (hkl) is the miller indices, a = b, and c are the lattice parameters. It is discovered that the examined films have ‘a’ and ‘c’ that are substantially identical to the standard value of TiO_2_ crystals with a = b = 3.785 Å and c = 9.513 Å, indicating that the films are crystalline [39]. The lattice parameters are slightly different than bulk, indicating that there is a stress in the doped films and that stress is produced due to some lattice distortion (shown in Table 3).

The crystallite size (D_s_) is an essential tool in crystallography as the sizes of the crystallites indicate whether the material is soft (small crystallites) or brittle (large crystallites). The size of D_s_ depends on nucleation and growth kinetics during deposition processing. Table 3 shows the variation of D_s_ of the films which were determined using the Scherrer formula (Eq. 5) [40]:

(5) $$D_{s}=\frac{k\lambda }{\beta \;\cos\;\theta }$$

where k (=0.9) is the Scherrer constant or form factor, λ = 1.54 Å wavelength of Cuk_α_, θ denotes the Bragg’s angle, and β is known as the full width at half maximum (FWHM). It is clear that β and D_s_ are inversely connected. In this study, the value of D_s_ for the pristine TiO_2_ film is small compared to doped films (except 8 at.%) and it is found that with increasing dopant concentration D_s_ is decreased gradually. This slight decrease in D_s_ suggests that doping of Cr ions can inhibit the growth of anatase crystals by increasing Cr-O-Ti bonds. Moreover, the decreasing trend of D_s_ also expresses the broad peaks in the XRD.

### 3.3 Dislocation density and lattice strain

Dislocation density (δ) is a measure of a crystal’s internal disorder that is calculated as the mean dislocation line length per unit crystal volume. A crystal structure with this kind of flaw is said to have a crystallographic defect. Its presence has a significant impact on the characteristics of the materials. Mathematically, it is a topological type of defect of crystal, and the value of δ increases with plastic deformation. Dislocation is caused by the different processes, including homogeneous nucleation, grain boundary initiation at the interface between the lattice and the surface, precipitates, scattered phases, and reinforcing fibers. Chen and Hendrickson determined δ and its relationship to the hardness of several silver crystals. They discovered that crystals with a greater δ value are harder [41]. Previously it was observed that the value of δ increased with strain (*ε*) while decreasing grain size, and finally, these parameters had attained saturation [42]. Using the following Eqs. (6), (7), the value of δ and *ε* of the films were calculated.

(6) $$\delta =\frac{1}{D_{s}^{2}}$$

(7) $$\varepsilon =\frac{\beta \;\cos\;\theta }{4}$$

**Fig. 5:**
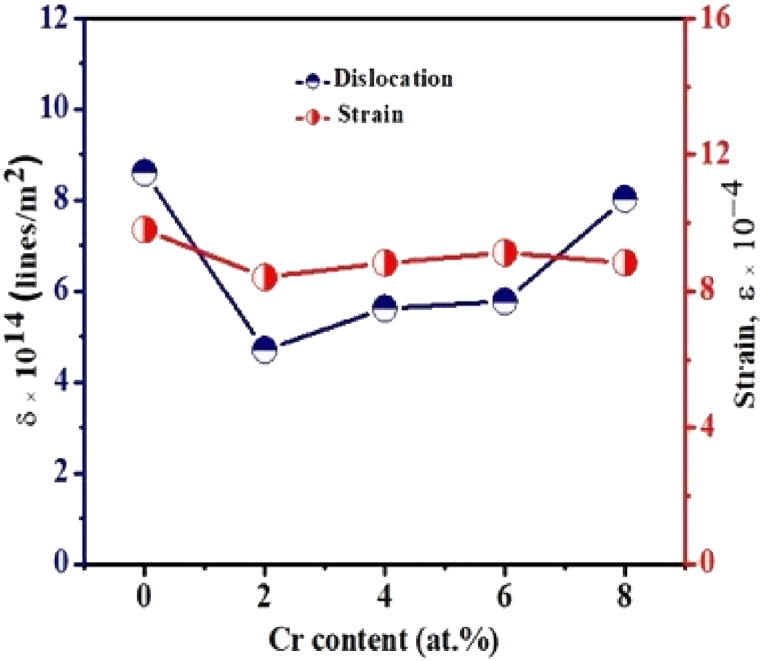
Change of dislocation density and lattice strain as a function of Cr content of pristine TiO_2_ and TiO_2_:Cr thin films.

The fluctuation of the dislocation density and lattice strain in relation to Cr concentration is seen in Fig. 5.

The values of δ and *ε* for the pristine TiO_2_ film are much higher than for Cr-doped films. The existence of low δ and *ε* values in doped thin films may be attributable to the movement of interstitial atoms from the grain boundary to the crystallites. Moreover, it is also noticed that the value of δ of the films decreased at first and then increased, which reveals the improvement of the crystallinity with proper Cr doping.

## 4 Optical evaluation

### 4.1 Absorbance

**Fig. 6:**
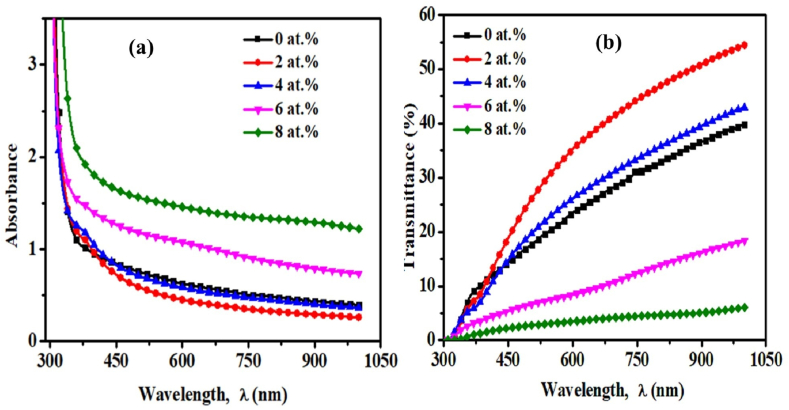
Change of (a) absorbance and (b) transmittance of pristine TiO_2_ and TiO_2_:Cr thin films as a function of λ.

Fig. 6(a) shows the absorbance of pristine TiO_2_ and TiO_2_:Cr thin films deposited on the glass substrate and it is discovered that the pristine TiO_2_ film is more transparent than doped films in the ultraviolet area. The absorbance edge is shifted from the ultraviolet area to the visible region, which is referred to as the redshift and is indicative of the band gap narrowing. In the visible range of the wavelength, absorbance decreases for 2 and 4 at.% and then increases for 6 and 8 at.% of Cr content. Previous studies also found an increasing trend of absorbance for other transition metal doped TiO_2_ semiconductors [43]. The strong absorbance edge is noticed below 400 nm, which may be responsible for attributing the ligand to metal charge transfer transition (O^2−^ to Ti^4+^) [44]. This finding also strongly shows that the interaction of the Cr atom with the TiO_2_ lattice changes the electrical characteristics of the Ti-oxide species, allowing light absorbance to shift from the ultraviolet to the visible spectrum. The transmittance of the films is shown in Fig. 6(b), which is the fraction of incoming electromagnetic power that is transferred through the films.

Transmittance data is evaluated by using Eq. (8):

(8) $$T=10^{-A}\times 100$$

where A is the absorbance and T is the transmittance. In the visible area, the films are very transparent. The transmission of the films begins to drop about 600 nm, probably due to the existence of Ti^3+^ states that produce d-d transitions [45]. It is noticed that 2 at.% TiO_2_:Cr film exhibits the highest transmission and after 2 at.%, the transmittance steadily declines, which may be related to an increase in photon scattering that is produced by crystal defects caused by doping. A similar observation was found for Zn-doped TiO_2_ thin films [46]. The decreased transparency (>10%) of the 8 at.% Cr-doped film may have resulted from the morphological and microstructural alterations caused by phase transition, as seen by the XRD patterns. Therefore, TiO_2_:Cr thin films are a viable choice for use in diverse optoelectronic and photovoltaic applications.

It has been observed that TiO_2_:Cr thin films have higher absorption of visible light compared to pristine TiO_2_. This enables these materials to harness a broader spectrum of sunlight for the applications in the photovoltaics. Moreover, the extension of the absorption spectrum into the visible range enhances the efficiency of TiO_2_:Cr thin films in optoelectronic devices, such as solar cells and LEDs by increasing their light absorption and photon conversion efficiency. It is also noted that Cr doping introduces defects in the TiO_2_ lattice, which may lead to increase carrier recombination rates. Thus a higher amount of Cr content can reduce the overall efficiency of TiO_2_:Cr in photovoltaic and photocatalytic applications.

### 4.2 Refractive index and extinction coefficient

To analyze insight into the optical behavior of the pristine TiO_2_ and TiO_2_:Cr films, we have to investigate the refractive index (η). It is a dispersion parameter that is closely connected to the electronic polarization of ions and the local field within any optical material. It is important to understand how a wave (of a given wavelength) is attenuated as it travels through a glossy medium since this may occur via a number of different processes, including the production of phonons, light generation, free carrier absorption, and scattering. Furthermore, the η of a semiconducting material (usually for hv < E_gap_) increases when E_gap_ decreases or vice versa [47,48]. The researcher has established various formulas to correlate η and E_gap_. Based on the atomic model, Moss has made the following relation between η and E_gap_

(9) $$\eta^{4}E_{gap}=K^{\prime }$$

where ‘K’ is a constant with 95 eV [40].

**Fig. 7:**
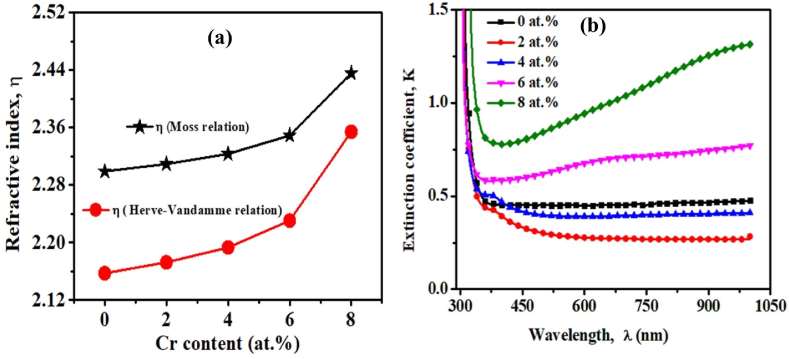
(a) Variation of refractive index (η) and (b) imaginary component of the refractive index or extinction coefficient (k) of pristine TiO_2_ and TiO_2_:Cr thin films as a function of the wavelength of photon.

Again, according to Herve-Vandamme [41], η can be calculated from the following equation:

(10) $$\eta =\sqrt{1+{\left(\frac{A}{E_{g}+B}\right)}^{2}}$$

where A and B are constants corresponding to 13.6 and 3.4 eV, respectively, Fig. 7(a) shows the variation of η that was determined using the above Eqs. (9), (10) for pristine TiO_2_ and TiO_2_:Cr thin films and presented their values in Table 4.

**Table 4:** E_gap_, and η for pristine TiO_2_ and TiO_2_:Cr thin films.

Cr:TiO_2_(x)	Energy, E_gap_ (eV)	Refractive index, η	
		Moss relation	H–V Relation
0	3.40	2.300	2.158
2	3.34	2.313	2.178
4	3.26	2.330	2.196
6	3.12	2.350	2.231
8	2.70	2.414	2.323

It is very clear that the investigated values of η for pristine TiO_2_ and TiO_2_:Cr films are consistent with the empirical data. This increasing trend of η with the decrease of E_gap_ occurs due to the incorporation of impurity atoms in the pristine TiO_2_ lattice. The imaginary part of the refractive index, which is also known as the extinction coefficient (k) is determined by the sum of the optical losses caused by absorption and photon scattering (Fig. 7(b)). Using the following relations (Eq. (11)), k was calculated [15].

(11) $$k=\frac{\alpha \lambda }{4\pi }$$

where photon wavelength is λ, and the absorption coefficient is α.

For a clear medium, k has a relatively tiny value, whereas for an absorbing medium, k has a significant value. It is found that the maximum k value is found for 8 at.% TiO_2_:Cr film, and the minimum k value is for 2 at.% TiO_2_:Cr film, which is the coexistence of the transmittance spectra. However, as a function of wavelength, k exhibits oscillatory behavior with a reduced value in the short wavelength range, as well as an anomalous dispersion with absorption bands in the visible part of the spectrum. Anomalous dispersion is noticed in substances that are not excessively opaque at the resonance frequency and it exhibits within the absorption band [49].

### 4.3 Dielectric study

The dielectric behavior of any material provides information regarding optical transition and phonon excitation within the material. It is a complex quantity consisting of the real part (ε_r_) and imaginary part (ε_i_), forming the relation *ε* = ε_r_ + ε_i_.

**Fig. 8:**
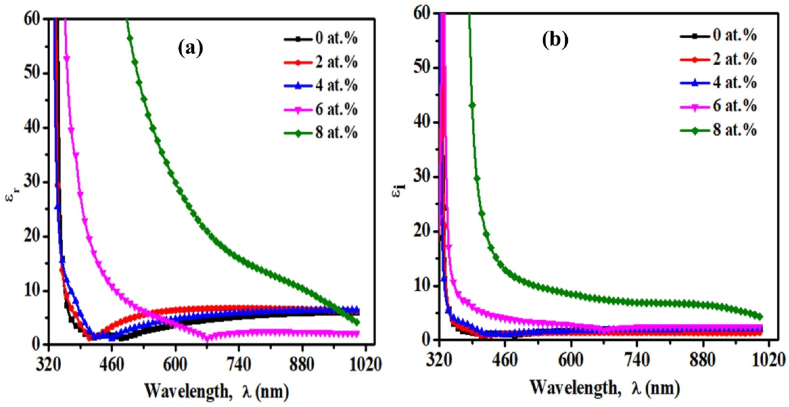
Variation of (a) the real component and (b) the imaginary component of the dielectric constant of pristine TiO_2_ and TiO_2_:Cr thin films as a function of λ.

The value ε_r_ denotes the property of slowing the speed of light within the film, whereas ε_i_ relates to the process of absorbing energy from the field because of dipole motion. The values of ε_r_ and ε_i_ are calculated using the following relation (Eq. (12)) [50].

(12) $$\varepsilon_{r}=\eta^{2}-k^{2}\;\text{and}\;\varepsilon_{i}=2\eta k$$

where η represents the wavelength-dependent refractive index and k represents the extinction coefficient. The exploration of the dielectric behavior of the deposited materials is depicted in Fig. 8, whereas the variation of ε_r_ with respect to frequency is denoted by Fig. 8(a), and the variation of ε_i_ is denoted by Fig. 8(b). In the region of visible and near-infrared, the pristine TiO_2_ film has a dielectric constant (real part) of 6, indicating that it is transparent at these wavelengths [51]. The values of both ε_r_ and ε_i_ are increased with increasing photon energy. Similar observations were investigated in previous studies [52]. Following a similar trend as the absorbance data, the value of ε_r_ and ε_i_ is increased with increasing Cr content.

### 4.4 Optical conductivity

The linear function that is related to current density in the electric field is defined as dynamical conductivity σ(q, ω). In the case of q→0, the σ(q, ω) becomes optical conductivity or σ_opt_(ω), and for ω→0, the conductivity is referred to as dc electrical conductivity σ(0). σ(0) describes the response of the longitudinal field of any system, and σ (ω) describes the transverse electric field. Optical conductivity has two parts; the real part and the imaginary part. The real part explains the dissipation of electromagnetic energy, and the screening of the applied field of the films is described by the imaginary part.

**Fig. 9:**
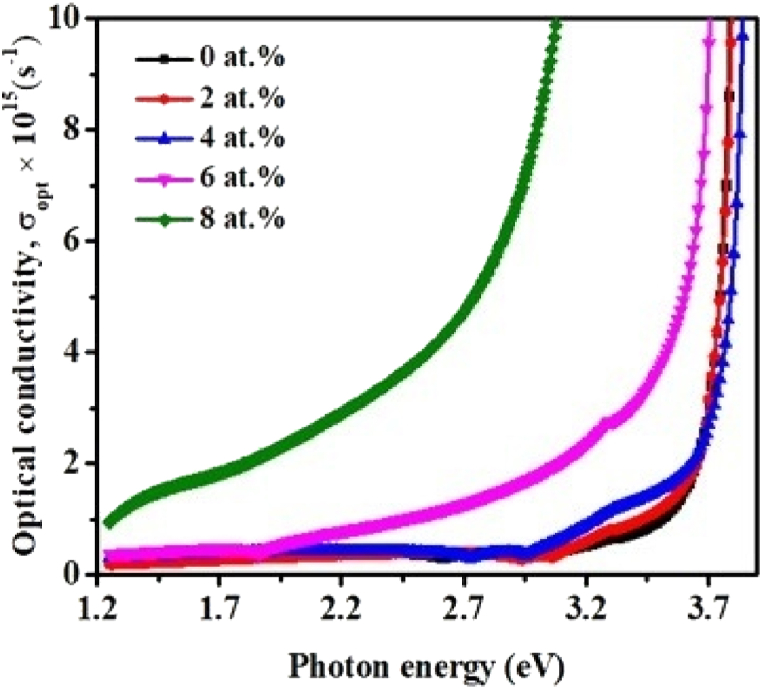
The dependence of the optical conductivity on the photon wavelength for pristine TiO_2_ and TiO_2_:Cr thin films.

σ_opt_ is highly dependent on α, η, E_gap_, k, and incident photon frequency. It is also a crucial parameter for studying the electronic states of any material. Fig. 9 depicts the optical conductivity that was determined by using Eq. (13) [53]:

(13) $$\sigma_{opt}=\frac{\alpha \eta c}{4\pi }$$

where c denotes the velocity of light. It is noticed that the value of σ_opt_ is higher at higher photon energy. This may be a result of the high absorbance of pristine TiO_2_ and TiO_2_:Cr thin films as well as the photon-excited electrons. In addition, photons of low energy cannot drive the hopping motion of charge carriers, resulting in a decrease in optical conductivity. It is also noticed that σ_opt_ increases somewhat with Cr doping, which can be attributed to charge carrier hopping between Ti^3+^ and Ti^4+^ ions. The dopant element Cr may generate oxygen vacancies, hence enhancing the hopping mechanism and resulting in an increase in the films' optical conductivity.

### 4.5 Optical band gap

The optical band gap (E_gap_) is an intrinsic property of any material and can be modified by doping impurity atoms in the lattice site of the material. For its measurement, electrons must be excited across the valence-to-conduction gap at a certain frequency. Above the basic absorption threshold, the optical transition is determined by the classical relation used to derive the bandgap energy (E_gap_) [54].

(14) $$\alpha h\nu =B{\left(h\nu \;–\;E_{gap}\right)}^{1/m}$$

where B represents the band-tailing parameter constant, hv is the photon energy, and m represents the inter-band transition, which is dependent on the crystalline or non-crystalline nature of the material. Depending on the values of m, the band gap can be categorized, where directly allowed, indirect allowed, directly forbidden, and indirect forbidden transitions are 2, 1/2, 3, and 3/2, respectively [55]. To determine the E_gap_ of the pristine TiO_2_ and TiO_2_:Cr films, (αhν)^2^ versus (hν) graphs were drawn. The linear fitting of (αhν)^2^ vs (hν) graphs has ensured the direct allowed bandgap (E_gap_) of the films, and a similar result was found in the previous study [19]. In this regard, the modified form of Tauc’s relation (Eq. (14)) can be written as like as Eq. (15),

(15) $${\left(\alpha h\nu \right)}^{2}=h\nu$$

**Fig. 10:**
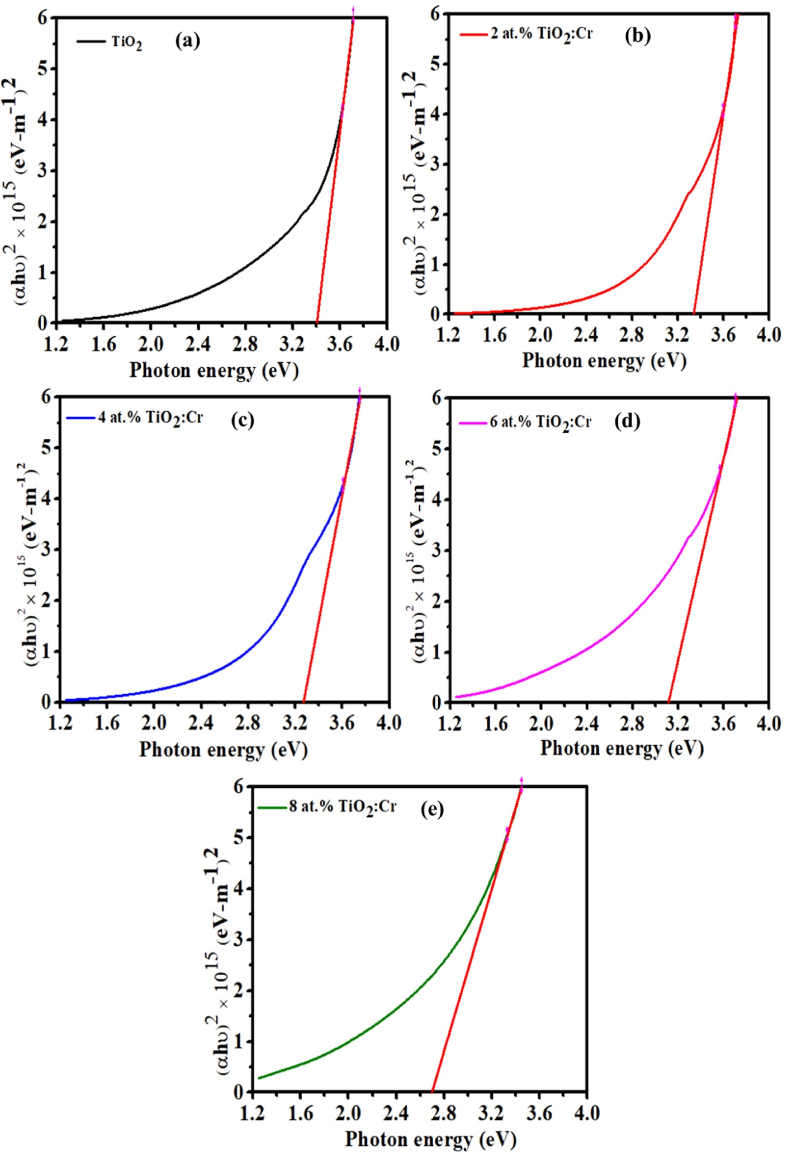
Optical band gap of (a) pristine TiO_2_, (b) 2 at.%, (c) 4 at.%, (d) 6 at.%, and (e) 8 at.% TiO_2_:Cr thin films.

Fig. 10(a–e) shows that the linear segments of the plots are extrapolated towards the absorption edge equal to zero (y = 0), which provides E_gap_ value for direct transitions. The E_gap_ of the films decreases from 3.40 to 2.70 eV (Table 4), indicating a redshift that may be attributable to the inclusion of Cr ions into the TiO_2_ lattice. This declining tendency of E_gap_ is a result of the introduction of new foreign atoms. Hajjaji et al., observed that the values of E_gap_ reduced from 3.33 to 1.48 eV when the Cr content increased from 0 to 17 at.% [56]. It is known that in pristine TiO_2_, the valence band is formed with O 2p states, and the conduction band is constructed with Ti 3d states [57]. In pristine TiO_2_ lattice, electronic transition takes place directly across the valence to the conduction band, but in Cr-doped films, unoccupied Cr^3+^ sd states are created, which can seize electrons. Moreover, oxygen vacancies are also created, and these states as well as oxygen vacancies, are connected to the reduction of the E_gap_ in the Cr-doped thin films [45,58].

Furthermore, the existence of oxygen vacancies causes pores, defects, or localized electronic states in the system’s bandgap. These extra oxygen vacancies raise the density of electrons within the energy gap region, causing E_gap_ to drop. Besides, Cr^3+^ ion is an acceptor impurity in the TiO_2_ lattice that generates energy levels close to the valence band edge. With increasing doping levels, the density of states of these dopants rises, resulting in a continuum of states that resemble energy bands and the E_gap_ effectively shrinks.

In a nutshell the optical properties of our studied samples focused the following investigations: Increased absorbance ensures that a greater proportion of incoming sunlight is captured by the material, resulting in enhanced efficiency in converting light energy to electricity within solar cells. Materials possessing elevated optical conductivity that effectively transform absorbed photons into electric current, thereby heightening the overall efficacy of solar cell performance. The determination of how proficiently a material absorbs incoming light, as influenced by its extinction coefficient, directly impacts the efficiency of energy conversion. Based on the outcomes of our optical analysis, there is promising potential for employing these findings in solar cell applications.

## 5 Temperature-dependent electrical properties

### 5.1 Resistivity

**Fig. 11:**
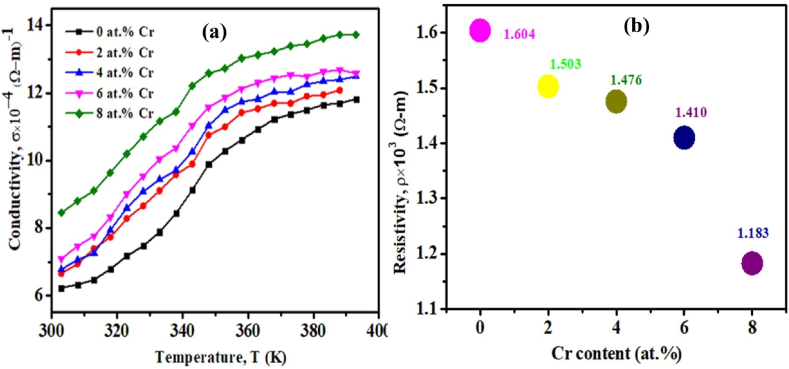
(a) Temperature-dependent conductivity of pristine TiO_2_ and TiO_2_:Cr thin films and (b) resistivity of pristine TiO_2_ and TiO_2_:Cr thin films at RT.

The temperature-dependence of the electrical conductivity (σ) of pristine TiO_2_ and TiO_2_:Cr thin film is presented in Fig. 11(a). Initially, with increasing temperatures, σ increases continuously. After temperature of 380 K, the output current increases very slowly, this might result in the reduced amount of ions mobility of the films.

It is known that thermally stimulated current can fill up the trap levels (available carried electrons above the Fermi-level E_F_), and then, at the time of heating, the thermal energy KT activates the trapped carriers, which take part in the transport phenomena either by hopping to other defect levels or via the conduction band [59]. The temperature at which carriers can be released is governed by the depth of the trap within the band gap. On the other hand, the progressive reduction of ρ with rising temperature indicates an increase in the mobility of charge carriers. Fig. 11(b) illustrates the electrical resistivity at RT for pristine TiO_2_ and TiO_2_:Cr films, and the corresponding resistivity is found at 1604, 1503, 1476, 1410, and 1183 Ω-m, respectively.

This decreasing trend of resistivity with increasing Cr content represents the better electrical conductivity, which is consistent with the increases in optical absorption. This reduction could also be explained by the fact that some of the Ti atoms have been replaced by Cr, which means that the shell isn’t completely full and has states close to the base of the conduction band. More conduction electrons will fill up these extra band states, lowering the resistivity of the films.

**Fig. 12:**
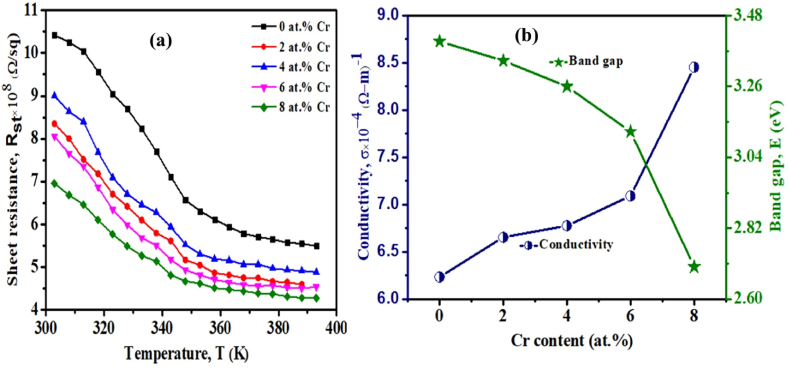
(a) Sheet resistance for different temperatures and (b) electrical conductivity and optical band gap for pristine TiO_2_ and TiO_2_:Cr thin films.

Fig. 12(a) depicts the sheet resistance (R_st_) of pristine TiO_2_ and TiO_2_:Cr thin films that were recorded for different temperatures. In the case of two-dimensional materials, calculating sheet resistance is a very important parameter. The sheet resistance of these two-dimensional deposited films is determined using the following formula,

(16) $$R_{st}=\frac{\rho }{d}$$

In Eq. (16), ρ is known as the temperature-dependent resistivity and d is the thickness of the pristine TiO_2_ and TiO_2_:Cr thin films. R_st_ gradually decreases with increasing temperature, and after 380 K temperature, it's value is almost constant following the trend of ρ due to the reduced amount of ions mobility. It is also noticed that the sheet resistance of the films decreases progressively as the Cr content increases, which might be ascribed to an increase in charge carrier concentrations in the films. The variation of E_gap_ and σ with Cr content is shown in Fig. 12(b) at RT. It has been discovered that the E_gap_ is reduced by the addition of Cr into TiO_2_ lattice. The higher-level inclusion of Cr has decreased the band gap of TiO_2_ films due to the entrance of acceptor levels in the forbidden band gap, which leads to an increase in σ. Essentially; transition metals may induce substantial changes in the electrical structure and E_gap_ value of the host crystalline materials. The addition of Cr dopant to the TiO_2_ lattice generates additional defect sites and excites more electrons across the valence-to-conduction gap [60], hence increases conductivity.

### 5.2 Activation energy

**Fig. 13:**
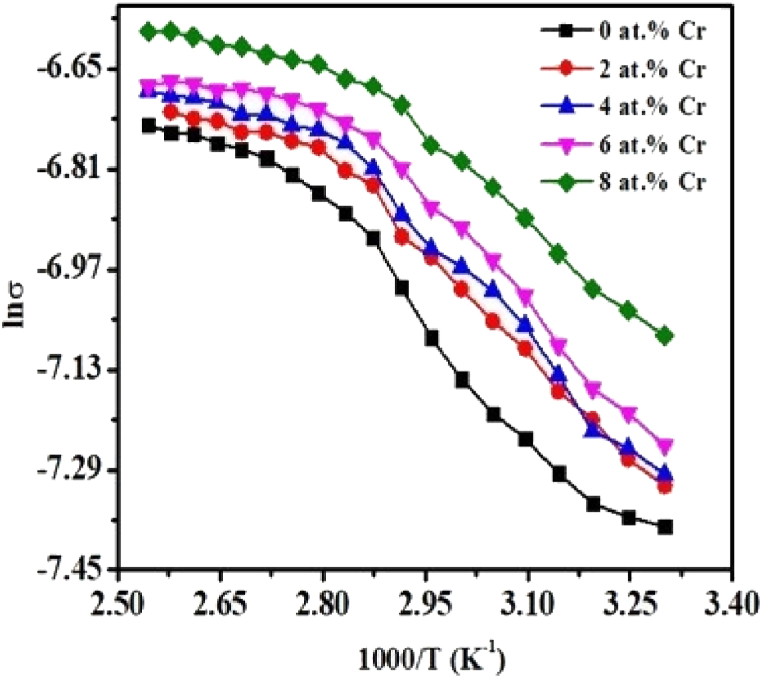
Plot of lnσ vs 1000/T of pristine TiO_2_ and TiO_2_:Cr thin films.

Fig. 13 expresses the plot of lnσ vs 1000/T for the pristine TiO_2_ and TiO_2_:Cr thin films which contains two regions of different conduction processes. Region I (303–363 K) and region II (363–393 K) correspond to activation energies E_I_ and E_II_, respectively. With increasing temperature, the conductivity increases. It is known that acceptor impurities can form energy levels near the valence band edge, and thermal energy can excite the carrier electrons. The increased amount of temperature pushes up a greater number of charge carriers to overcome the activation energy barrier that can take part in the electrical conduction mechanism. The temperature-dependent conductivity can be written as

(17) $$\sigma =\sigma_{o}\;\exp\;\left(\;\frac{-\Delta E}{{2k}_{b}T}\right)$$

Where σ_0_ is the pre-exponential factor, T denotes absolute temperature, k_b_ (=8.617×10^−5^ eVK^−1^) is the Boltzmann constant, and ΔE denotes the amount of the activation energy that means the energy difference between acceptor levels and the valence band.

Eq. (17) appears as

(18) $$\ln\;\sigma =-\frac{\Delta E}{{2k}_{b}T}+\ln\;\sigma_{o}$$

**Fig. 14:**
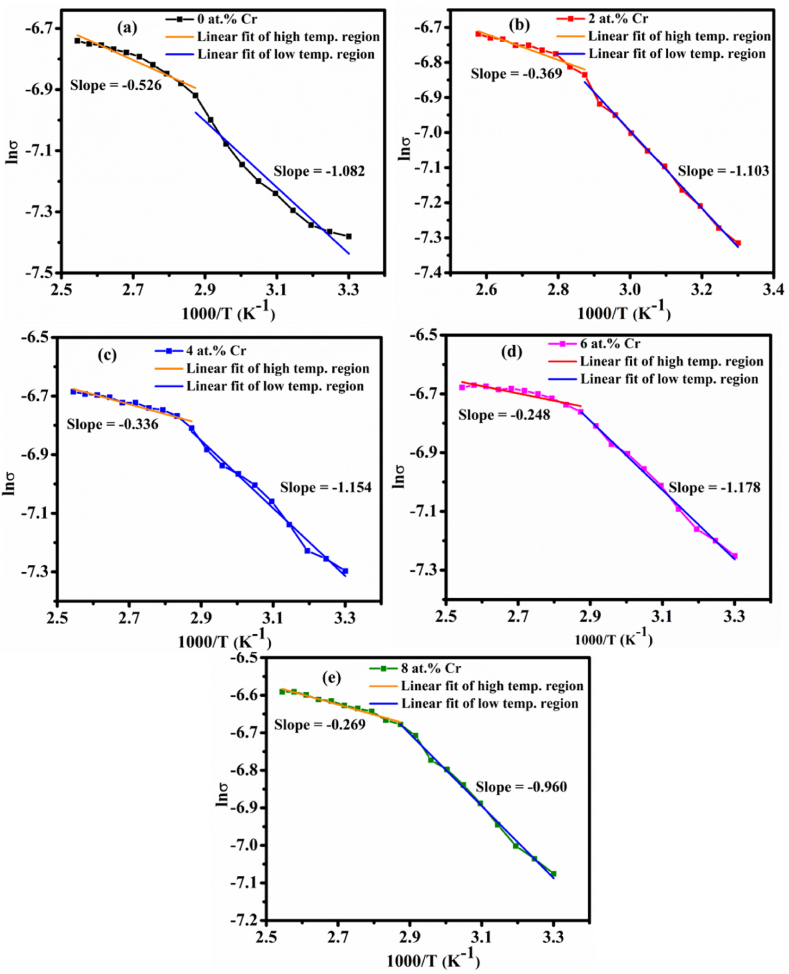
Linear fitting of lnσ vs 1000/T graphs for (a) pristine TiO_2_, (b) 2 at.%, (c) 4 at.%, (d) 6 at.%, and (e) 8 at.% TiO_2_:Cr thin films.

Eq. (18) is a similar equation of the straight line (y = mx + c) corresponding slope is $\;-\frac{E}{{2k}_{b}T}\text{.}$ The activation energy is obtained by equating the graphical slope of the films with the slope of Eq. (18) (Fig. 14). The first slope of each film (high temperatures region) is linked to the band conduction or activation of carriers from the acceptor levels to the valence band, and the second slope (low temperatures region) is related to the polaron process. The activation energy of Cr acceptors ranges from 13 to 61 meV, while prior research determined it to be 45–50 meV [61]. The activation energy $\Delta E_{I}$ increases progressively with increasing Cr concentration up to 6 at.%, indicating that a high amount of energy is required for the carrier hopping process in the film at temperatures between 303 and 363 K. Also, the value of $\Delta E_{II}$ drops as the Cr concentration rises, indicating that small polaron mobility is increased by a decrease in hopping activation energy (shown in Table 5). In addition, the use of an ionic oxide semiconductor with minimum crystallinity, a rough surface, and a low level of the disorder increased the electrical conductivity [62].

**Table 5:** Resistivity at 303 and 393 K temperatures and activation energy of pristine TiO_2_ and TiO_2_:Cr thin films at low temperature and high-temperature region.

Cr:TiO_2_ (x)	Resistivity (Ω-m)		Activation energy	
	303 K	388 K	${\Delta E}_{I}$ (eV)	${\Delta E}_{II}$ (eV)
0	1604	855	0.056	0.027
2	1503	828	0.057	0.019
4	1476	807	0.060	0.017
6	1410	789	0.061	0.013
8	1183	729	0.050	0.013

## 6 Conclusions

By using the spray pyrolysis deposition technique, TiO_2_ thin films were successfully synthesized, and the homogeneous incorporation of Cr^3+^ in the place of Ti^4+^ was confirmed by EDX analysis. Appearing fibers in the 6 and 8 at.% Cr-doped films indicate that higher dopants could lead to form fiber growth. Up to 6 at.% Cr-doped films show the anatase crystal structure, and anatase-rutile mixed phase for 8 at.% Cr-doped film gives an idea about the limiting level dopant of Cr ion in the SPD technique. The absorption edge is shifted towards a higher wavelength, and the corresponding decrease in band gap values represents a redshift. The resistivity is decreased with increasing temperature as well as Cr content. The decreasing trend of the optical bandgap and the increasing trend of the optical conductivity indicate that our studied compositions can be good candidates for solar cell applications.

## Funding

This research did not receive any specific grant from funding agencies in the public, commercial, or not-for-profit sectors.

## Data statement

The data used for this article is derived from the experiments. These are not available online.

## Data availability

Data will be made available on request.

## CRediT authorship contribution statement

**Abdul Ahad:** Writing – original draft, Methodology, Investigation, Formal analysis, Data curation. **Jiban Podder:** Writing – review & editing, Visualization, Validation, Supervision, Resources, Project administration, Data curation, Conceptualization. **Tusar Saha:** Validation, Methodology, Investigation, Formal analysis, Data curation. **Hari Narayan Das:** Software, Investigation, Data curation.

## Declaration of competing interest

There is no conflict of interest in this manuscript from any person or from any Institution.
